# An analysis of Liberia's 2007 national health policy: lessons for health systems strengthening and chronic disease care in poor, post-conflict countries

**DOI:** 10.1186/1744-8603-7-37

**Published:** 2011-10-10

**Authors:** Patrick T Lee, Gina R Kruse, Brian T Chan, Moses BF Massaquoi, Rajesh R Panjabi, Bernice T Dahn, Walter T Gwenigale

**Affiliations:** 1 Massachusetts General Hospital, 55 Fruit Street, Boston, Massachusetts, USA; 2 Harvard Medical School, 25 Shattuck Street, Boston, Massachusetts, USA; 3 Tiyatien Health, Hospital Road, Zwedru, Grand Gedeh County, Liberia; 4 Clinton Health Access Initiative, 383 Dorchester Avenue, Suite 400, Boston, Massachusetts, USA; 5 Ministry of Health and Social Welfare, Capital Bypass, Monrovia, Liberia

## Abstract

**Background:**

Globally, chronic diseases are responsible for an enormous burden of deaths, disability, and economic loss, yet little is known about the optimal health sector response to chronic diseases in poor, post-conflict countries. Liberia's experience in strengthening health systems and health financing overall, and addressing HIV/AIDS and mental health in particular, provides a relevant case study for international stakeholders and policymakers in other poor, post-conflict countries seeking to understand and prioritize the global response to chronic diseases.

**Methods:**

We conducted a historical review of Liberia's post-conflict policies and their impact on general economic and health indicators, as well as on health systems strengthening and chronic disease care and treatment. Key sources included primary documents from Liberia's Ministry of Health and Social Welfare, published and gray literature, and personal communications from key stakeholders engaged in Liberia's Health Sector Reform. In this case study, we examine the early reconstruction of Liberia's health care system from the end of conflict in 2003 to the present time, highlight challenges and lessons learned from this initial experience, and describe future directions for health systems strengthening and chronic disease care and treatment in Liberia.

**Results:**

Six key lessons emerge from this analysis: (i) the 2007 National Health Policy's 'one size fits all' approach met aggregate planning targets but resulted in significant gaps and inefficiencies throughout the system; (ii) the innovative Health Sector Pool Fund proved to be an effective financing mechanism to recruit and align health actors with the 2007 National Health Policy; (iii) a substantial rural health delivery gap remains, but it could be bridged with a robust cadre of community health workers integrated into the primary health care system; (iv) effective strategies for HIV/AIDS care in other settings should be validated in Liberia and adapted for use in other chronic diseases; (v) mental health disorders are extremely prevalent in Liberia and should remain a top chronic disease priority; and (vi) better information systems and data management are needed at all levels of the health system.

**Conclusions:**

The way forward for chronic diseases in Liberia will require an increased emphasis on quality over quantity, better data management to inform rational health sector planning, corrective mechanisms to more efficiently align health infrastructure and personnel with existing needs, and innovative methods to improve long-term retention in care and bridge the rural health delivery gap.

## Introduction

Globally, non-communicable diseases (NCDs) are responsible for an enormous burden of deaths and economic loss, much of which could be prevented through concerted action on intermediate risk factors such as smoking, diet, and physical inactivity [1,2]. In Sub-Saharan Africa, urbanization and adoption of Western lifestyles is driving an emerging epidemic of cardiovascular, chronic respiratory, and oncologic disease [3-5]. This rise of chronic disease in Africa alongside the unfinished agenda of communicable, malnutrition-related, and maternal, newborn, and childhood disease has been called a 'double burden, ' requiring a 'double response' that emphasizes strengthened primary care systems capable of providing comprehensive acute, episodic, and chronic care [6,7].

But this formulation oversimplifies the textured landscape of chronic disease in Africa. There are at least three overlapping but distinct chronic disease epidemics in Africa, corresponding to the urban rich, the urban poor, and the rural poor. The epidemiology of chronic disease and therefore the necessary interventions differ substantially across these three populations [8]. In poor rural populations, for example, cardiovascular disease is prevalent but is only rarely the result of atherosclerosis and coronary disease [9,10]. Instead, cardiomyopathy results from infections, pregnancy, alcohol, or malignant hypertension [11,12]. Strategies to reduce the usual risk factors (smoking, diet, lack of exercise) in poor African populations could miss their mark. Similarly, mental health is an enormous, grossly underappreciated problem [13]. Treatment gaps for depression, epilepsy, substance abuse, and stroke approach 100% in many of these settings [14-17], despite the existence of cost-effective packages of mental health care that could be integrated into primary care systems [18,19].

A crippling knowledge gap exists in poor areas, such that little is known about, and therefore little is done to prevent and treat the "long tail of chronic disease" that perpetuates suffering, constrains development, and creates conditions for insecurity and conflict in the world's poorest areas [20,21]. The United Nations General Assembly Special Session in September 2011 (the results of which were not known at the time of writing) therefore presents both an historic opportunity to advance the global NCD agenda and a very real risk that the rural poor will be left behind. Concerted action on tobacco control and other cardiovascular risk factors will save millions of lives and billions of dollars in the aggregate, but it may also widen inequalities between poor, rich, rural, and urban populations. Similar rigor, enthusiasm, and action should be invested in solutions that apply to poor populations whose problems are so often unmeasured, unknown, and ignored.

The experience of a country such as Liberia, emerging from war with a bold vision to become "an international model of post-conflict recovery, " may therefore be of particular value to the global community [22]. In this case study, we chart the early reconstruction of Liberia's health care system from the end of conflict in 2003 to the present time, highlight challenges and lessons learned from this initial experience, and reflect on the principles and policies Liberia incorporated in its 2011 National Health Policy and Plan. We close with thoughts on the way forward for chronic disease in Liberia.

Our goal is to answer the question: given Liberia's experience to date, what are the emerging lessons for addressing chronic diseases in a poor, post-conflict country through a strategy of innovative health financing and health systems strengthening?

## Methods

We conducted a historical review of published and gray literature on Liberian policies affecting health systems strengthening and chronic disease prevention and treatment, including plans for poverty reduction and health and social welfare planning in the post-conflict period. Documents were primarily country level policies sourced from the Government of Liberia Ministry of Health and Social Welfare. Key documents included the Poverty Reduction Strategy, the 2007 National Health Policy, the Basic Package of Health Services, and the 2011 National Health Policy and Plan. The documents were considered reliable as they represent direct sources describing the post-conflict recovery and health system in Liberia. We reviewed published literature on Liberian health services and outcomes, as well as health systems for chronic disease management in post-conflict settings. Measurable results of the initial post-conflict policies were also reviewed to characterize their early impact.

In order to describe the preceding conditions and early impact of the 2007 National Health Policy, we reviewed general economic indicators such as gross domestic product, measures of income inequality, total health spending, and out-of-pocket health spending. We selected these indicators based on the following observations: (i) general economic indicators have been associated with a variety of health outcomes including maternal mortality [23]; (ii) inequality in income distribution and other development factors such as education explain even more variation in mortality [24]; (iii) per person or percent GDP expenditure is correlated with health outcomes such as maternal mortality and child mortality [23,25]; and (iv) high out-of-pocket payments may induce poverty and lead to further negative health consequences [26]. Furthermore, we reviewed health indicators that are particularly affected by conflict including under-five and maternal mortality, overall mortality, and existing health infrastructure including workforce distribution and measures of primary care access. Given the significant interdependencies of health and socioeconomic status, the above range of indicators were necessary to provide a reasonable picture of Liberia's challenges and progress in the post-conflict period.

## After the War

By the time Liberia emerged from civil war in 2003, fourteen years of brutal conflict had ruined Liberia's economy, infrastructure, health system, and the health and education of its people. Of Liberia's 550 pre-war health facilities, only 354 facilities (12 public hospitals, 32 public health centers, 189 public clinics, 10 private health centers, and 111 private clinics) were functioning by the end of 2003. Eighty percent of these were managed by non-governmental organizations (NGOs) and faith-based organizations (FBOs) [27]. The nation performed a rapid assessment of the total clinical workforce including private, NGO, and government workers. They estimated the workforce at 3, 107 persons: 168 physicians, 273 physician assistants (PAs), 443 registered nurses (RNs), and more than 1, 000 nurse aides. In addition to being small in number, the workforce was mismatched to the country's needs. There were too few physicians and PAs, and most health workers were located in the capital city. Destruction of health training institutions left just one school with appropriate resources to train health care workers [28]. Non-health education was comparably devastated. About 70 percent of school buildings were partially or wholly destroyed, and over half of Liberian children and youth were estimated to be out of school. A whole generation of Liberians had spent more time in war than in school.

The war ruined Liberia's economy. By the 2005 elections, average income in Liberia was just one-fourth of what it had been in 1987, and just one-sixth of its level in 1979. In nominal terms, GDP per capita was $160 in 2005 [29]. By 2003, unemployment and underemployment were extremely high, with ex-combatants, returning refugees, and internally displaced persons struggling to find work. At that time, refugees returning to their farms faced a lack of seeds, fertilizers, tools, and in some cases uncertain land tenure. Government finances collapsed in tandem with the economy. Government revenue fell to less than $85 million USD per year between 2000 and 2005, translating into public spending of only about $25 USD per person per year, one of the lowest levels in the world. In 2010, prior to debt cancellation by the International Monetary Fund and the World Bank, Liberia's total debt was approximately $4.9 billion USD, equivalent to 800% of its GDP and 3, 100% of its exports.

## Revitalizing the Health System

Against this daunting backdrop, President Ellen Johnson Sirleaf and her administration created bold new policies with the goal of transforming Liberia into "an international model of post-conflict recovery." The Poverty Reduction Strategy (PRS) was created to move toward rapid, inclusive, and sustainable growth and development during the period 2008-2011 [30]. The focus of the PRS was broad, with intended improvements ranging from better roads to a revitalized health system.

Building on the PRS, the Liberian Government created the National Health Policy (NHP) in 2007 in order to "improve health and social welfare status and equity in health" [22]. Key features of the 2007 NHP included:

• Committed to decentralization, with County Health Teams given greater authority over county health facilities;

• Acknowledged three tiers of care - primary, secondary, and tertiary;

• Suspended user fees at the primary and secondary level, though user fees remained at the tertiary level; and

• Committed Liberian government to progressively increase health spending to eventually meet the Abuja target of 15% of the national budget.

The 2007 NHP also outlined the Basic Package of Health Services (BPHS) that would be provided without charge at clinics and hospitals regardless of geographic location [31]. The BPHS was based on principles of decentralization and primary health care, and focused on a limited set of entitlements including two chronic diseases: mental health (encompassing depression, epilepsy, substance abuse, and gender-based violence) and HIV/AIDS (estimated nationwide prevalence of 1.5% in 2007). As is the case across Africa and in similar non-African settings such as Haiti [32], HIV/AIDS serves as the template of chronic disease in Liberia, about which the most is known, and from which lessons may be applied to health systems planning and service delivery for other chronic diseases. The BPHS was a consistent, measureable package of services that enabled facilities to be funded by different donors through a competitive bidding process while minimizing inconsistencies in the services provided [33]. Overall, the 2007 NHP and BPHS were consistent with the observation that a focus on primary care initiatives and infrastructure is an effective method for health system strengthening [34].

Aligning the efforts of a large number of health actors with the NHP and BPHS presented a significant challenge. To meet this challenge, an innovative financing mechanism called the Health Sector Pool Fund was established in March 2008 through a Joint Financing Agreement between the Liberian Government and its international partners. The Pool Fund operated under the oversight of the Ministry of Health and Social Welfare (MoHSW) and had four main objectives: (i) to help finance priority unfunded needs within the NHP; (ii) to increase the leadership of the MoHSW in the allocation of resources; (iii) to reduce transaction costs associated with managing multiple different donor projects; and (iv) to take the first steps toward sector budgeting and sectoral budget support [35]. Under the MoHSW's stewardship, the Pool Fund grew from an initial $8 million USD in 2008 to over $35 million USD in 2010. By 2010, all major international partners except the U.S. Government were channeling their contributions through this mechanism, and the Pool Fund had facilitated decentralization of BPHS implementation to two of Liberia's sixteen counties through a competitive process involving three-way partnerships between Liberian County Health Teams, international NGOs, and local NGOs.

## Progress and Challenges

By 2010, these strategies had yielded significant improvements in Liberia's economy, health system, and the health of its people, though major challenges remained.

### Economy and Health Financing

Economic indicators improved significantly. GDP grew 7.1% in 2008 with continued growth estimated in the 7-11% range [36]. Inflation dropped from double digits to 7.4% in 2009 [36]. International Monetary Fund and World Bank requirements for debt forgiveness were met in June 2010 [36], cancelling $4.6 billion USD of the country's staggering $4.9 billion USD debt. The cancellation of the debt has placed the country in a better position to attract new loans to finance badly needed infrastructural improvements.

Following the roadmap laid out by the 2007 NHP required significant increases in health care spending. In 2008, total health and social welfare expenditure reached over $100 million USD, equivalent to $29 USD per person per year or 15% of GDP up from 9% of GDP in 2003 [37]. Due to delays and limited administrative capacity among County Health Teams, the majority of funding was still managed by a combination of NGOs and the central MoHSW [33]. External donors and households accounted for a large proportion of expenditure at 47% and 35% respectively, while government spending accounted for 15%. Although state funding for health continued to increase, there was a growing funding gap caused by the departure of NGOs that had entered the sector at the end of the conflict.

Despite increased public expenditures, out-of-pocket expenses remained high at 35% of health costs [37]. This level of personal expenditure was a disproportionate burden for the poor. Data from the 2008 Community Health Seeking Behavior Survey indicated that although a majority of households (64%) lived below the poverty line, each household spent approximately $10 USD per person per year on health [38]. The poorest 20% of the population spent as much as 17% of their annual income on health.

### Health Infrastructure and Human Resources

Measurable gains in infrastructure and human resources also occurred. In 2010, the aggregate number of functioning health facilities met the National Health Plan target [39] and resulted in a dramatic increase in access to primary health care, with each health facility now serving an average of 5, 500 people as of 2009, down from 8, 000 in 2006 [40]. In addition, the 2010 facility accreditation process found that 80% of functioning government facilities met the minimum standards for provision of the BPHS [39]. In 2009, a national human resources census recorded 9, 196 health and social welfare workers, up from 3, 107 in 2003 [41].

Though aggregate targets for number of health facilities were met, and 80% of these facilities met minimum BPHS standards, 41% of all households (15% urban and 66% rural) did not have ready access to a health facility [42]. The NHP envisaged an assessment of rehabilitation and construction needs based on utilization, population, geographic access, cost, and other socioeconomic factors, but this long-term assessment had not been carried out. By including rapid targets for renovation or construction of health facilities in the NHP and PRS in the absence of a thorough needs assessment, a significant amount of capacity and resources were committed to targets that were not based on evidence or patient preference [33,43,44]. By 2010, many clinics fell outside catchment criteria established by the MoHSW; over 50% of government clinics were serving catchment populations smaller (40%) or larger (11%) than the established criteria [45]. Given the difficulty in applying a standard package of services to a diverse spectrum of facilities, rural-urban disparities remained a particular challenge. No national formula existed for determining the level of resource allocation to counties based on population, utilization, and access criteria [46].

The MoHSW established a human resources unit that is unique among government ministries [35]. Among the human resource unit's achievements was the revitalization of nurse and mid-level provider training. The Martha Tubman School of Midwifery reopened in Grand Gedeh County; the Esther Bacon School of Nursing and Midwifery in Lofa County reopened; and the Tubman Medical Institute of Medical Arts in Monrovia was renovated. New skill sets were also developed. For example, the MoHSW created a job classification for trained and credentialed mid-level primary care providers to serve as mental health clinicians [33].

### Health and Chronic Disease

The health of the population clearly suffered in the conflict, but with economic and health systems development, health indicators were improving. Life expectancy had risen from 48 years in 1990 to 54 years in 2000 to 58 years in 2009 [29]. Infant mortality fell from 165 per 1000 in 1990 to 133 per 1000 in 2000 to 80 per 1000 in 2009 [29]. Under-five child mortality fell from 247 per 1000 live births in 1990 to 198 per 1000 in 2000 to 112 per 1000 in 2009 [29]. Maternal mortality had nearly doubled, however, rising to 994 per 100, 000 live births in 2007 from 550 per 100, 000 live births in 2000 [47], highlighting the inadequate coverage of safe delivery and surgical services, especially in rural areas, during this period.

With regard to chronic diseases for which data are available, gains in HIV/AIDS outpaced gains in mental health. Though an infectious disease, HIV/AIDS manifests as a chronic disease from the perspective of health systems, requiring a continuity patient-provider relationship over time; benefiting from strategies to promote adherence and retention in care; and manifesting complex interactions with other co-morbid conditions. Across Africa, low-income countries have significantly greater experience in HIV/AIDS care than in any other chronic disease, making this condition the most useful lens through which to assess a low-income country's capacity and derive lessons learned for chronic disease care.

In 2006, only 742 HIV positive patients were enrolled in care and treatment programs in Liberia. By 2010, the National AIDS Control Program (NACP) had scaled up HIV/AIDS service delivery points from 20 to 162 HIV counseling and testing sites, from 2 to 142 prevention of mother-to-child transmission sites, and from 5 to 24 HIV care and treatment sites. This rapid scale-up resulted in a nine-fold increase in HIV/AIDS care and treatment delivery, with 3, 907 patients receiving antiretroviral therapy (ART) and a total of 6, 804 patients enrolled nationwide.

Adherence and retention in care for HIV/AIDS patients on ART remained a significant challenge, however, with an average lost to follow-up rate at 12 months of 27% at HIV treatment centers across the country [48]. Fortunately, innovative strategies to improve long-term retention of ART patients had begun to emerge. Liberia's first and largest rural treatment center, for example, achieved long-term retention rates significantly higher than the national average through a community-based approach that included directly observed therapy (DOT) and integrated social support, organized around a backbone of trained and salaried community health workers [49,50]. Jointly administered by the County Health Team and a local NGO, this strategy closely mirrored the DOT-HAART approach pioneered and validated elsewhere [51]. Observational data from this treatment center document a 60% higher retention and survival rate among HIV patients on ART followed by CHWs compared to ART patients without a CHW [49]. In 2010, the Liberian National AIDS Control Program, the Global Fund for AIDS, Tuberculosis, and Malaria, and a local NGO created a partnership to pilot the DOT-HAART model, delivered by salaried CHWs and supervised by MoHSW clinicians, at twenty HIV care and treatment centers across the country [52].

Regarding mental health, significant progress was made in characterizing the striking burden and multiple barriers to mental health care. A nationwide household survey in 2008 provided valuable insight into the prevalence of psychiatric illness and its relationship to the war [17]. Surveyors found that 44% of participants had symptoms of post-traumatic stress disorder, 40% met criteria for major depressive disorder, 11% reported suicidal ideation, and 6% reported a prior unsuccessful suicide attempt. Only 2.4% of former combatants and 7.8% of former non-combatants reported sufficient access to local mental health services. 97.5% of participants reported significant barriers to health care. The two most prevalent barriers were 'lack of payment ability' (underscoring the significant burden of out-of-pocket expenses among the poor, despite the absence of user fees) and 'health care too far away' (consistent with the finding that 41% of all Liberian households and 66% of rural households are located more than one hour away from the nearest health care facility).

The MoHSW responded vigorously to the challenge of untreated mental disorders affecting nearly half of all Liberians. Minister of Health and Social Welfare Walter Gwenigale vetoed the first version of the BPHS because it failed to include mental health, arguing that such an omission would ignore one of the most significant barriers to Liberia's health and development [53]. Following the inclusion of mental health as one of six focus areas in the 2007 BPHS, Liberia developed a National Mental Health Policy (NMHP) in 2009 and a Basic Package of Mental Health Services (BPMHS) in 2010, making it one of only a handful of countries in Africa with a dedicated national mental health policy. The NMHP and BPMHS outlined staffing standards, standardized diagnostic evaluation, and specified the menu of services that should be offered at each facility level, from clinic to tertiary hospital.

These positive developments in central planning for mental health contrast sharply with relatively little progress in implementation and delivery at the county and facility level. In 2010, MoHSW efforts were underway to upgrade mid-level primary care providers to serve as mental health clinicians, but mental health services were still being delivered on an *ad hoc *basis, as evidenced by the decision of the national drug service not to procure amitriptyline (the only antidepressant on Liberia's national formulary) since there was so little demand (Liberia MoHSW Lead Procurement Officer, personal communication, August 10, 2010). Nevertheless, pilot service projects were emerging in the rural areas. One such initiative, spearheaded by a local NGO in partnership with the Grand Gedeh County Health Team, had developed standardized protocols and a home-based care model led by CHWs, and had enrolled several hundred depression and epilepsy patients in care [54]. In 2010, the Grand Gedeh County Health Team, partnering with an international NGO and a local NGO, received a two-year grant through the Pool Fund mechanism to scale up its mental health intervention across sixteen other primary care facilities in Grand Gedeh County. Outcomes data from this collaboration are forthcoming.

## Lessons Learned and Way Forward

There were many lessons learned from the implementation of the 2007 NHP and BPHS. Building from these lessons, the MoHSW convened a broad range of domestic and international partners from September 2010 through June 2011 to create a National Health Policy and Plan (NHPP) for the next ten years with the stated objective "to reform and manage the sector to effectively and efficiently deliver comprehensive, quality health and social welfare services that are equitable, accessible, and sustainable for all people in Liberia."

In the following section, we discuss six lessons with specific relevance to health systems and chronic diseases, including recommendations relevant to health sector policy, planning, financing, implementation, delivery, and evaluation. We also describe the nascent 2011 NHPP's response to the lessons that emerged from the 2007 NHP and BPHS experience. The six lessons and Liberia's responses in the 2011 NHPP are summarized in Table 1.

**Table 1:** The way forward for chronic disease in Liberia

Lessons Learned	Way Forward
**1. The 2007 National Health Policy's 'one size fits all' approach met aggregate planning targets but resulted in significant gaps and inefficiencies throughout the system**.	• Fully implement the legal and administrative framework necessary for decentralization of the health sector; • Emphasize a Primary Health and Social Welfare Care approach that encompasses decentralization, community empowerment, and inclusive partnership; and • Base resource allocation criteria on the services to be provided and the size, density, and geographic location of the catchment population.

**2. The innovative Health Sector Pool Fund proved to be an effective financing mechanism to recruit and align health actors with the 2007 National Health Policy**.	• Establish a National Health and Social Welfare Financing Policy to build on the Health Sector Pool Fund experience; and • Progressively increase government contribution to the health and social welfare sector, towards its Abuja commitment of 15% of total government expenditures.

**3. A substantial rural health delivery gap remains, but it could be bridged with a robust cadre of community health workers integrated into the primary health care system**.	• Revise the National Strategy and Policy for Community Health to improve integration of Community Health Workers (CHWs) into all levels of the health system; and • Strongly consider paying CHWs, given the critical role they will be asked to play and the well-documented challenges of volunteerism.

**4. Effective strategies for HIV/AIDS care in other settings should be validated in Liberia and adapted for use in other chronic diseases**.	• Apply lessons learned from HIV/AIDS care to other chronic disease care (e.g., task-shifting, community-based care, reducing or eliminating out-of-pocket costs to patients); and • Test innovative methods to improve long-term retention in care (e.g., linking clinical and social services, adapting CHW home-based care to mental health disorders).

**5. Mental health disorders are extremely prevalent in Liberia and should remain a top chronic disease priority**.	• Continue to prioritize mental health in the 2011 National Health Policy and Plan; and • Implement basic mental health services at the health center and community level.

**6. Better information systems and data management are needed at all levels of the health system**.	• Implement a National Health Information System; and • Explore and deploy low-cost mobile technologies to improve community-based data collection and care delivery.

Six lessons learned from the first iteration of Liberia's National Health Policy from 2007-2010, and Liberia's response in the 2011 National Health Policy and Plan.

### Lesson #1 - The 2007 BPHS 'one size fits all' approach failed to respond to distinct local needs

The fixed criteria and guidelines for facilities, staffing, and services provided by the BPHS resulted in a 'one size fits all' approach that failed to respond to communities' distinct needs and preferences. A rational, flexible approach to resource allocation and service delivery, informed by a nationwide situational analysis, is needed to ensure more efficient and effective health care delivery at all levels.

At the facility level, rigid criteria were applied to facility distribution, staffing levels, and provision of drugs. The final package of services represented an average set of requirements for all facilities both large and small. Instead of a 'one size fits all' approach, large facilities staffed with numerous teams of assorted skills should be planned for large populations with easy access to them, while a different package should be designed to promote multiple service delivery points while keeping costs down for sparsely-settled populations [33,40].

Rigid staffing criteria were established, and like facility catchment criteria, these norms were inappropriate for small clinics. Clinics were penalized under BPHS if 'understaffed, ' so clinics were incentivized to fully staff even if not necessary to meet clinical demand. This resulted in a mismatch of worker mix to local health and service delivery needs, with a general shortage of physicians and physician assistants, and a relative excess number of nurses and unskilled workers. Furthermore, some workers were under-qualified for their cadre. For example, 44% of nurses lacked the level of education required by their professional association [33]. Retaining skilled workers in rural areas was especially challenging, thus exacerbating the geographic mismatch. Weak management structures - particularly as decentralization was slow to materialize - contributed to all of these staffing difficulties.

The need for a flexible response has particular relevance for chronic diseases. The epidemiology, patient preferences, and optimal interventions for chronic disease in Liberia are likely to differ between rich urban, poor urban, and poor rural populations. In other African nations, the burden of chronic disease is growing most rapidly among the urban poor [55]. Higher rates of hypertension among urban compared to rural populations have been measured in the neighboring countries of Ghana [56] and Cameroon [57], attributed to increased rates of physical inactivity, adoption of Western diets, and increased body mass index. In contrast, ischemic heart disease and its risk factors are extremely rare in rural African populations [58]. It may be possible that aggressive public health campaigns aimed at tobacco cessation and control, reduced salt intake, or regular physical activity could help deter the epidemic from reaching currently unaffected populations.

### 2011 NHPP Response

Moving away from a 'one size fits all' policy, the 2011 NHPP will institute a Primary Health Care (PHC) approach that encompasses decentralization, community empowerment, and inclusive partnership. The MoHSW will fully implement the legal and administrative framework necessary for decentralization of the health sector - a process that was intended in the 2007 NHP but not begun in earnest. More autonomy and funding will be transferred from the central MoHSW to the County Health Teams (which currently manage less than 1% of the financial resources in the sector) [33]. Furthermore, the tiered system for health delivery (community, primary, secondary, and tertiary) will be solidified. At each operational level, the intended structure will be clarified and staff and citizens empowered to make decisions that affect their health.

At the facility level, the MoHSW will move from inflexible prototypes and staffing requirements to a standards-based approach. Criteria for allocating, staffing, and supplying health and social welfare facilities will be based on services to be provided and the size, density, and geographic location of the catchment population. For example, health posts staffed by a single certified midwife, RN, or PA will be built to serve remote rural areas where patients otherwise would have to travel more than one hour by foot to reach health care services. The MoHSW also plans to institute more robust hardship remuneration schemes to attract and retain health workers in rural areas.

### Lesson #2 - The Pool Fund was an effective and efficient financing mechanism to recruit and align health actors with the 2007 NHP and BPHS

Liberia's Health Sector Pool Fund had a transformative impact on the health sector. It helped increase annual health expenditures to $29 USD per person per year, enabling decentralization of the BPHS to a majority of public health facilities by 2010. This resulted in targeted system and service improvements (informed by the BPHS accreditation process) that successfully increased BPHS accreditation rates from 35% in 2009 to 80% in 2010, exceeding national targets by 10% [30].

The Pool Fund also strengthened country ownership and coordination between government, local NGOs, and international NGOs by empowering the MoHSW to contract service provision to partners aligned with the goals of the 2007 NHP. The advent of the Pool Fund required the creation of robust financial transparency mechanisms, such as the strengthening of the MoHSW's Office of Financial Management, enhancing the MoHSW's capacity to effectively administer other major grants and funding partnerships.

Some limitations persist, including the lack of civil society participation on the Pool Fund's Steering Committee and the absence of major financial contributions from the U.S. government. In addition, the Pool Fund stops short of the "capacity to disburse resources beyond [the] public system and beyond [the] health sector when this represents appropriate and cost-effective approach to improve health outcomes, " which has been proposed as a key feature of health system financing [59]. Despite these limitations, Liberia's Health Sector Pool Fund provides a valuable example for governments of other low-income countries seeking to increase direct budgetary support, strengthen country ownership, and expand financial transparency within their health sectors.

Liberia's innovative public sector financing mechanism should be rigorously evaluated, with successful aspects broadly disseminated and implemented across sufficiently mature Ministries of Health that face similar challenges.

### Relevance to World Health Organization "Health Systems Financing: The Path to Universal Coverage" Framework

Liberia's experience with the Pool Fund has particular relevance for low-income countries seeking to implement the framework for action proposed by the WHO in its 2010 World Health Report, "Health Systems Financing: The Path to Universal Coverage" [60]. While significant challenges remain, the Pool Fund offers one approach to achieving several key recommendations from the WHO report, in particular: (i) pay for health in ways that do not deter access to services; (ii) consolidate funding pools; and (iii) use resources more efficiently and equitably. On this final point in particular, the Pool Fund excelled through its contributions to improved central governance and accountability, reduced fragmentation across the health system, new opportunities for strategic purchasing of and contracting for health services, and overall reduction of waste.

Furthermore, the Pool Fund stands out as a 'best-in-class' example of the international community fulfilling key components of the agenda described in the WHO report, specifically: (i) helping Liberia reach the required level of overall financing; (ii) supporting Liberia's health plan rather than imposing external priorities; (iii) channeling funds through the institutions and mechanisms crucial to universal coverage; (iv) supporting local efforts to use resources more efficiently; and (v) reducing duplication and fragmentation in international aid efforts.

### 2011 NHPP Response

Over the next 10 years, the MoHSW will build on this early experience by establishing a National Health and Social Welfare Financing Policy to guide financing decisions. As donor contributions inevitably decline, the Liberian Government plans to progressively increase the share it apportions to the health and social welfare sector, towards its Abuja commitment of 15% of total government expenditures. Other innovative financing strategies such as insurance and other forms of risk-pooling and pre-payment will be considered as a means of increasing social protection in light of high out-of-pocket payments from individuals. Predictable, effective, transparent, and decentralized means to channel support through the Government's national systems will be developed.

### Lesson #3 - The substantial rural health delivery gap could be bridged with a robust community health worker-oriented strategy

A substantial rural health delivery gap remains, with more than two-thirds of households located outside of facility catchment areas; a significant mismatch between available workforce and local health and service delivery needs; and limited referral and supervision capacity. A corps of Community Health Workers that is equipped, trained, well supported, and recognized as a formal cadre within the County Health Teams can link dispersed populations to services and facilities at reasonable cost, and should form the backbone of Liberia's rural health delivery strategy.

While reliable cost-effectiveness data are lacking, preliminary costing exercises from other countries such as Rwanda suggest that coverage of rural populations using community health workers can be achieved for as little as $3 USD per person per year (M. Rich, personal communication, January 25, 2011) or 7% of total health expenditures [61]. A major Doris Duke Initiative is currently funding a cost-effectiveness analysis of Rwanda's model of comprehensive primary care facilitated by universal access to trained and salaried CHWs [61].

In Liberia, community health was intended to make up a large proportion of the health sector and fill in the gap where facilities were lacking, but community health volunteers (CHVs) were poorly trained, poorly motivated, and difficult to retain. The aspiration of Liberia's 2008 National Strategy and Policy for Community Health Services - envisioning a range of high quality primary care services delivered by teams of well-supervised community volunteers - was poorly matched to the requirement that CHVs be 'unsalaried volunteers' [62]. Furthermore, significant delays in decentralization and BPHS implementation meant that CHVs were in practice poorly supervised and lacked the necessary access to referral medical services. The experience in Nimba County, which reported a disappointing 0% retention rate after two years among CHVs working with patients living with HIV/AIDS (M. Badio, MoHSW Monitoring and Evaluation Officer, personal communication), is emblematic of this mismatch of expectation and underinvestment. The illogic of expecting teams of paid medical professionals in health facilities and teams of unpaid, poorly supervised volunteers in the community to delivery the same package of health services at comparable quality has now been widely recognized (B. Chan, personal communication, January 28, 2011).

Examples from other resource-constrained settings have demonstrated compelling results when CHWs are equipped, trained, well supported, and recognized as formal members of the health team [51,63]. In Liberia, this approach has been implemented with preliminary success in HIV/AIDS and mental health and this care model is now being piloted at twenty HIV care and treatment sites nationwide [49]. Rigorous evaluation is needed to assess the feasibility, impact, and cost-effectiveness of this approach.

### 2011 NHPP Response

Bridging the rural-urban gap will be achieved in large part by implementation of the PHC approach as detailed above. In the context of current limitations in resources and skilled personnel, the Liberian Government also recognizes that a healthy and effective cadre of CHWs can extend the effective reach of each physical facility at lower cost and will be essential to the functioning of the health system as a whole. The MoHSW is therefore revisiting the current National Strategy and Policy for Community Health. The MoHSW plans to integrate CHWs more closely with all levels of the health system in order to improve timely referrals and perform important tasks such as monitoring treatment and delivering bednets and vaccinations.

Training and supervision of CHWs will also be enhanced. For example, the current supervisor for CHWs at the health facility level is generally a nurse-aide level employee. In the next iteration of the policy, this supervisor will change to a more skilled employee, such as an RN, PA, or Environmental Health Technician. These health officers will be accountable for outcomes in their catchment areas and report to the County Health Officer, who is in turn accountable to the central MoHSW.

In response to challenges in motivating and retaining CHWs, Liberia plans to revisit the issue of remuneration for CHWs. Given anticipated investments in training and supervision for CHWs and integration of CHWs into all tiers of the health care system, it will be necessary to explore innovative methods including remuneration in order to motivate and retain these valuable workers.

### Lesson #4 - Effective strategies for HIV/AIDS care should be validated and adapted for use in other chronic diseases

Liberia has begun scaling up care and treatment for HIV/AIDS, but loss to follow-up among ART and pre-ART patients remains a major challenge. Effective management of patients who qualify for ART requires a lifetime commitment by both patients and providers to a complex multi-drug treatment regimen with significant side effects. Examples of programs from other parts of Africa demonstrate that features of a chronic care model - efficient patient flow, excellent care pathways, ready access for caregivers to critical historical information, and feedback of patient and population outcomes to clinical providers - can strengthen quality of HIV treatment [64].

The health workforce is severely constrained across Africa, yet numerous HIV/AIDS programs have demonstrated how task-shifting from physicians to other health providers and community health workers can be effective in treating this particularly human resource-intensive chronic disease [65]. Task-shifting - an approach widely endorsed by the World Health Organization and others - relies on simplified, evidence-based protocols, robust training with ongoing support, and quality-assured, low-cost drugs and diagnostics. The task-shifting approach validated for HIV/AIDS is directly relevant to non-communicable chronic diseases and primary health care in general. Indeed the 'Patient-Centered Medical Home' model at the forefront of the U.S. healthcare reform effort derives from the same core principles of optimal stewardship of health resources, with each cadre of worker making full use of their competency and training so that the overall system delivers better health and better care at lower cost [66-70].

Yet despite effective and low-cost interventions and increased availability of ART in resource-poor settings such as Liberia [71-77], long-term patient retention remains a significant challenge. Strategies shown to be effective in improving retention in care and HIV/AIDS outcomes in other settings include eliminating co-payments or medication costs, personal counseling, and providing social services such as nutrition support or reimbursement for transportation [78]. These methods can be cost-effective and should be validated in HIV/AIDS populations in Liberia and then adapted to other chronic diseases.

### 2011 NHPP Response

The 2011 NHPP will apply lessons learned from HIV/AIDS care to inform design and implementation of effective care for other chronic diseases. Emphasizing task-shifting to foster optimal stewardship of health resources is at the center of this effort. Pilot initiatives have already demonstrated improved outcomes in HIV/AIDS care with a task-shifted strategy and integrated clinical and social services [49], and have begun to adapt this model to epilepsy and depression care [54]. Data from recent MoHSW-led efforts to scale up this model are forthcoming.

Efforts to maximize task-shifting should also be enhanced by the MoHSW's newly established National Human Resources for Health and Social Welfare Policy and Plan. This policy includes flexible staffing criteria and measures to improve workforce performance, such as linking recruitment, career development, standardized remuneration, and hardship incentives to service distribution and service delivery priorities. Furthermore, training and accreditation programs will be created to upgrade the skills of active health workers. By maximizing workforce performance, the 2011 NHPP should enhance efforts to adapt task-shifting models from HIV/AIDS to other chronic diseases.

### Lesson #5 - Mental health disorders are extremely prevalent in Liberia and should remain a top chronic disease priority

Liberia's mental health experience exemplifies the knowledge gap that cripples rational health sector reform in poor countries around the world. Whereas the influential Global Burden of Disease (GBD) Report estimated unipolar depression deaths in Liberia at 0.1 per 100, 000 population and disability-adjusted life years (DALYs) at 612 per 100, 000 population (conservative estimates based on the absence of reliable data), the 2008 national survey reported a depression prevalence of 40% among Liberian adults, with 11% of adults reporting suicidal ideation and 6% of adults reporting a prior unsuccessful suicide attempt [17,79]. While these data do not allow a direct comparison of deaths, DALYs, or prevalence, they do suggest a large burden of disease that was missed in the GBD Report and revealed in the national survey. This kind of underestimation is a systemic problem. In the aggregate, these large underestimates result in the patently inaccurate characterization of mental health problems as relatively uncommon in low-income countries compared to middle- and high-income countries (the reverse is probably true) [79]. Since it is the known problems that attract greater international attention and funding, global mental health remains largely neglected and left off of international and national agendas [80].

Fortunately, national-level needs assessments such as the Liberian 2008 mental health survey [17] are feasible and can powerfully redirect policy and action - witness Minister of Health and Social Welfare Gwenigale's veto of the initial BPHS (based in part on preliminary findings of the 2008 study) and the subsequent inclusion of mental health in the BPHS, the development of the National Mental Health Policy and Basic Package of Mental Health Services, and the funded scale-up of an innovative mental health program under the Pool Fund mechanism [31,35].

Mental health disorders are extremely prevalent in Liberia, obstruct economic development, and heighten the risk of renewed violence. Mental health should therefore remain a top chronic disease priority for Liberia over the next ten years. Other poor, post-conflict countries in Africa likely have similarly massive, undocumented burdens of mental health problems. Recognizing this 'elephant in the room' could have transformative implications for countries' health sector reforms and the global agenda for chronic diseases.

### 2011 NHPP Response

Momentum for improving mental health care will continue to build with the 2011 NHPP. In 2010, the Ministry of Health and Social Welfare produced the Basic Package of Mental Health Services (BPMHS) that called for a significant expansion of mental health mid-level providers at multiple tiers of the system. To that end, the MoHSW is developing a job classification for the mental health clinician, an intensively trained and credentialed mid-level care provider. Furthermore, as part of its approach to Primary Health Care, the MoHSW plans to introduce basic mental health services at more clinics and health centers (at least 20% of all facilities) over the next decade. Finally, the establishment of a functional referral system should help ensure the throughput of patients with severe or complicated mental health disorders from the lowest to the highest levels of the health care system.

Going forward, Liberia's experience in bridging delivery and quality gaps in mental health could inform care for other chronic diseases and primary care in general. The primary access barriers to mental health care - poverty, distance from clinic, lack of transport, and cost burden [17] - are relevant across the spectrum of acute, episodic, and chronic care. Solutions to these challenges, which may include CHW-oriented strategies in rural areas or integrated clinical and social services as discussed above, could be usefully applied in the future to cardiovascular or cancer care. As was the case in Haiti with improved HIV/AIDS care, it is also possible that targeted efforts in mental health could win public confidence and thereby improve uptake of other priority services that are sensitive to patient preference, such as vaccination programs, family planning, and obstetric care [63].

### Lesson #6 - Better information systems and data management are needed at all levels of the health system

The need for improved information systems to enable operational improvement and rational health sector planning is clearly evident. Many aspects of care delivery including community-based care, decentralization, and hospital referral information remain undocumented and unavailable to health providers or policymakers. The paper-based data that do get collected tend to remain fragmented between consultants and various working groups, and are not often available in a form that can be absorbed and used [40]. Standardized methods of data collection, and ideally a robust national health database, would help policymakers make informed decisions as well as enable ongoing quality improvement at the facility and county levels. Low-cost technologies including mobile phone-based applications should be considered as a method to improve care and reporting from CHWs whose work occurs away from facilities. Mobile devices also offer opportunities for decision support to help health workers adhere to standardized protocols, and could provide additional supervision and accountability while improving quality of care.

The 2007 NHP recognized the need to establish a strong Health Management Information System. Significant progress has been made in this area, namely in the areas of staff recruitment and training, equipment procurement, revision of reporting tools (including a move towards electronic reporting), and the creation of a National Monitoring and Evaluation Policy in 2009 [81]. Standardized methods of data collection, and ideally a robust national health database that reflects the WHO concept of a National Health Information System (NHIS), would help policymakers make informed decisions as well as enable ongoing quality improvement at the facility and county levels [82].

### 2011 NHPP Response

The 2011 NHPP aims to move Liberia closer to the WHO's NHIS framework. With a strong data infrastructure and committed international partnership, Liberia could generate invaluable and sorely lacking comparative effectiveness data, helping to define what constitutes effective prevention and treatment of chronic diseases in poor, post-conflict settings. This process is beginning with a baseline survey of non-communicable diseases, conducted by the MoHSW in concert with the WHO. Covering five counties in Liberia, this survey is expected to conclude in 2011.

Liberia's very real resource constraints will require staged implementation of new services at both the county and facility level. If this sequential implementation could be randomized, and the data management infrastructure was in place, Liberia would be in a unique position to ethically test at-scale interventions in a randomized, controlled fashion in populations with substantial unmet need. The rigorous knowledge generated in this setting could be of immense value to the global community. With bold partnerships and the necessary external support, the dismantling of Liberia's health system during the war could be transformed into one of its greatest assets.

## Conclusion

Little is known about the optimal health sector response to chronic diseases in the poorest areas of the world. Liberia's experience in strengthening health systems and health financing overall, and addressing HIV/AIDS and mental health in particular, provides a relevant case study for international stakeholders and policymakers in other poor, post-conflict countries seeking to understand and prioritize the global response to chronic diseases.

During the tenure of the first National Health Policy from 2007 to 2010, significant progress was made, particularly in aligning disparate health actors through the Health Sector Pool Fund and achieving rapid scale-up in measurable aggregate health outputs such as facilities and health workers. Major challenges remain, including mismatch of outputs to needs, low quality of services and worker competencies, and a significant rural health delivery gap. Early experience with HIV/AIDS and mental health points to opportunities to validate and adapt lessons learned in other settings for the effective care of these and other chronic diseases in Liberia. The way forward for chronic diseases in Liberia will require an increased emphasis on quality over quantity, better data management to inform rational health sector planning, corrective mechanisms to more efficiently align health infrastructure and personnel with existing needs, and innovative methods to improve long-term retention in care and bridge the rural health delivery gap.

Liberia's poverty could be of great value to the world. A primary health care system is being built from the foundation up in a relatively small country under the thoughtful stewardship of a disciplined government with well-aligned international and local partners. Could Liberia help define what constitutes effective prevention and treatment of chronic diseases in poor, post-conflict settings? The answer will largely depend on the capacity of the international community to 'turn the world upside down' [83] and establish common cause with Liberia, bolstering its reconstruction efforts while benefiting from new ideas and innovations.

## Competing interests

The authors declare that they have no competing interests. PTL, GRK, BTC, and RRP are affiliated with Tiyatien Health. MBFM, BTD, and WTG are affiliated with the Liberian Ministry of Health and Social Welfare.

## Authors' contributions

PTL, GRK, BTC, MBFM, and RRP participated in the design of the case study and helped to draft the manuscript. BTD and WTG helped to draft the manuscript. All authors read and approved the final manuscript.

**Figure 1:**
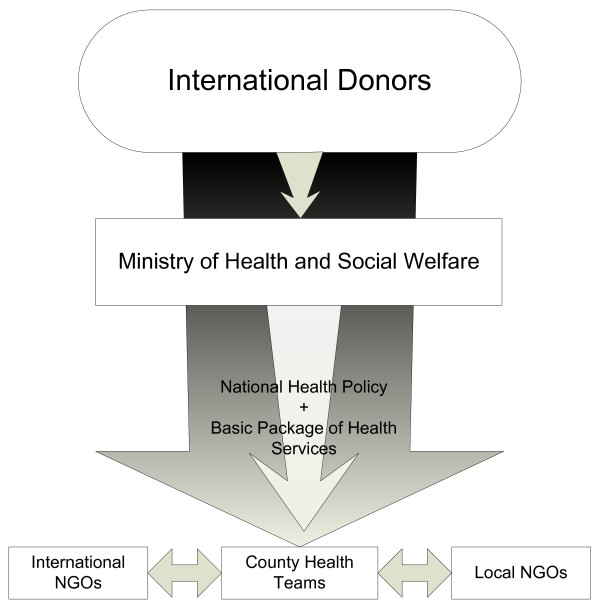
**The Liberian Health Sector Pool Fund**. The Liberian Health Sector Pool Fund is a multi-donor trust fund, administered by the Liberian Government with participatory oversight from donors and civil society partners, that accomplishes two principal goals: (i) strengthening the administrative capacity of the Liberian government while reinforcing good governance and accountability, and (ii) aligning local and international health actors with the National Health Policy and Basic Package of Health Services.
